# Neoadjuvant trastuzumab deruxtecan (T-DXd) with response-directed definitive therapy in early stage HER2-positive breast cancer: a phase II study protocol (SHAMROCK study)

**DOI:** 10.1186/s12885-024-11851-4

**Published:** 2024-01-17

**Authors:** Gavin P. Dowling, Sinead Toomey, Philip Bredin, Imelda Parker, Eibhlin Mulroe, Jacinta Marron, Olivia McLoughlin, Ausra Teiserskiene, Colm Power, Anne Marie O’Shea, Megan Greally, Patrick G. Morris, Deirdre Duke, Arnold D. K. Hill, Bryan T. Hennessy

**Affiliations:** 1 Royal College of Surgeons in Ireland (RCSI) University of Medicine and Health Sciences, 123 St Stephen’s Green, Dublin 2, Ireland; 2 Beaumont RCSI Cancer Centre, Beaumont Hospital, Dublin, Ireland; 3 Cancer Trials Ireland, Dublin, Ireland

**Keywords:** Breast cancer, Trastuzumab deruxtecan, HER2-positive, Neoadjuvant, Pathological Complete Response

## Abstract

**Background:**

The current standard of care in the neoadjuvant setting for high-risk HER2-positive (HER2 +) breast cancer is to combine systemic chemotherapy with dual HER2 blockade, trastuzumab and pertuzumab. Targeted therapies have significantly improved outcomes for patients with HER2-positive breast cancer. To improve treatment-associated toxicity, chemotherapy-sparing approaches are currently being investigated. Trastuzumab deruxtecan (T-DXd) is an HER2-directed antibody–drug-conjugate (ADC) with promising results in the metastatic setting for HER2-positive breast cancer. The SHAMROCK study investigates neoadjuvant T-DXd in early stage HER2-positive breast cancer, using pathological complete response (pCR) rate as the primary endpoint.

**Methods:**

This is a phase II open-label, single arm, adaptive multi-centre trial of T-DXd in the neoadjuvant setting in stage 2–3 HER2-positive breast cancer. Eligible patients will receive 5.4 mg/kg of T-DXd intravenously every 3 weeks for up to 6 cycles. A repeat biopsy will performed after 2 cycles for the RNA disruption index (RDI) score assessment. According to their likelihood of pCR, as determined by the RDI score, patients will either undergo 4 or 6 cycles of T-DXd prior to imaging. Patients with imaging complete response (iCR) after either 4 or 6 cycles will proceed to surgery. Patients who do not achieve iCR will either undergo further systemic therapy or proceed to surgery.

**Discussion:**

The SHAMROCK study is a chemotherapy-sparing approach to curative intent treatment, investigating neoadjuvant T-DXd. We hypothesise that neoadjuvant T-DXd will have a high pCR rate and be associated low toxicity in early stage HER2-positive breast cancer.

**Trial registration:**

EudraCT Number: 2022–002485-32; ClinicalTrials.gov identifier: NCT05710666; Cancer Trials Ireland study number: CTRIAL-IE 22–01.

## Background

Human epidermal growth factor 2 (HER2) is over-expressed and/or amplified in 15–20% of all breast cancers. HER2-targeted therapies, such as the monoclonal antibody trastuzumab, have significantly improved clinical outcomes for patients with HER2-positive (HER2 +) breast cancer. The current standard of care in the neoadjuvant setting for resectable high-risk HER2 + breast cancer is to combine chemotherapy with dual HER2 blockade, trastuzumab and pertuzumab, for a synergistic anti-tumour effect [1, 2]. Pathological complete response (pCR) after neoadjuvant therapy is associated with favourable survival outcomes [3], and can be used to individualise adjuvant systemic therapy. In patients with residual disease at surgery, improvements in disease-free-survival (DFS) have been seen with the addition of adjuvant trastuzumab-emtansine (T-DM1), an antibody–drug conjugate (ADC), as demonstrated in the KATHERINE trial [4].

Systemic chemotherapy is associated with significant short and long term side effects, including but not limited to alopecia, fatigue, gastrointestinal symptoms, life-threatening cytopenias and infections, infertility and secondary malignancies in later life. In the KRISTINE trial a chemotherapy-sparing approach with neoadjuvant T-DM1 combined with pertuzumab showed promising results, with a favourable toxicity profile [5]. However, this regimen has not been incorporated into routine practice due to a higher incidence of locoregional progression events prior to surgery. The lack of available biomarkers to guide pre-operative therapy by identifying patients in whom chemotherapy can be safely omitted is an area of unmet need [5, 6].

Trastuzumab deruxtecan (T-DXd) is an ADC combining an anti-HER2 antibody, a tetrapeptide-based cleavable linker and a topoisomerase I inhibitor payload [7]. The efficacy of T-DXd for advanced or metastatic HER2-positive breast cancer after progression on T-DM1 was established in the DESTINY-Breast01 trial [8] and confirmed in the randomised phase 3 DESTINY-Breast02 [9] trial. The rate of grade 3 or higher adverse events in the DESTINY-Breast03 trial, with a median treatment duration of 14.3 months, was 52.1% in a cohort of patients with pre-treated metastatic breast cancer that had progressed on trastuzumab and a taxane [10]. By way of comparison to a population of fit and treatment-naive patients with locoregional disease in the breast and axilla only, the rate of grade 3 to 4 adverse events in the chemotherapy arm of the KRISTINE trial was 64.4% [5].

The ribonucleic acid (RNA) disruption index (RDI) score is a molecular test based on the analysis of RNA disruption, a qualitative and quantitative alteration of the ribosomal RNA of tumour cells that correlates with chemotherapy response in primary breast cancer [11, 12]. The RDI score has also been shown to predict pCR after as early as one cycle of neoadjuvant chemotherapy in patients with HER2-positive breast cancer [13, 14]. To optimise pCR rates, the RDI score will be used as a biomarker for on-treatment tissue, together with breast imaging, to guide the use of pre-operative chemotherapy.

Therefore, we hypothesise that neoadjuvant T-DXd will have a high pCR rate and be associated with low toxicity in the early stages of HER2-positive breast cancer.

## Methods

### Trial design

**Fig. 1:**
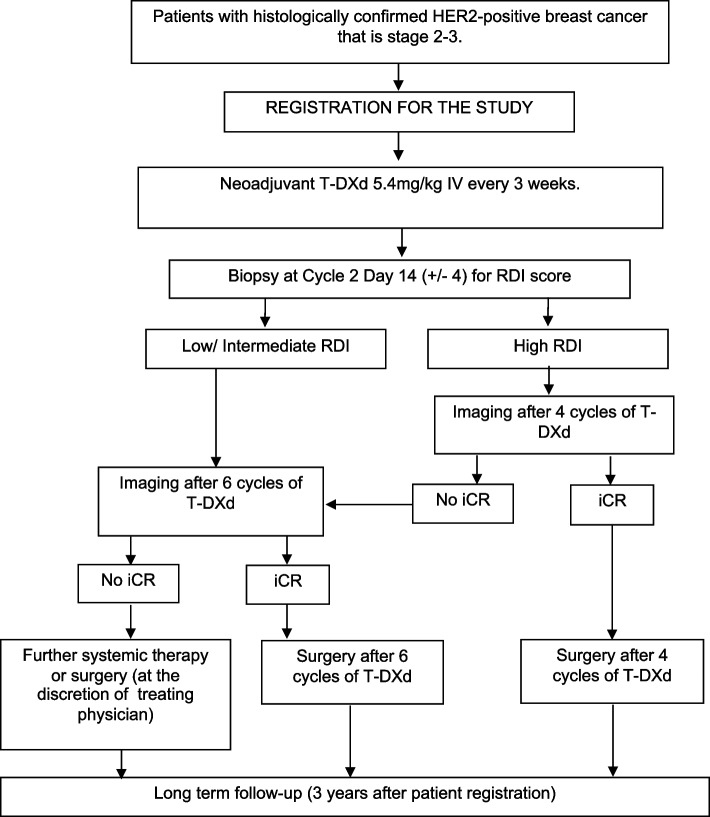
Study Schema of SHAMROCK study. Abbreviations: *RDI* RNA disruption index, *T-DXd* trastuzumab deruxtecan, *iCR* imaging complete response

The SHAMROCK study is a phase 2 open label, single-arm, adaptive multicentre trial. Patients with early stage (stage 2–3) HER2-positive breast cancer are treated with neoadjuvant T-DXd every 3 weeks for up to 6 cycles. A repeat biopsy after 2 cycles (at cycle 2 day 14) of T-DXd is performed for the RDI score assessment. As a safety measure patients will undergo clinical examination before each cycle of T-DXd to enable early identification of on-treatment locoregional progression. If progression is seen, then T-DXd will be stopped, and the patient taken off study treatment. In the absence of early progression, those patients with a high chance of pCR based on the RDI score undergo repeat breast imaging after four cycles of T-DXd. Patients who have a high chance of pCR based on the RDI score proceed to surgery after four cycles of T-DXd if they also have imaging complete response (iCR) at that point. Other patients who have a high chance of pCR based on the RDI score but iCR is not attained after four cycles or who have a low/intermediate chance of pCR based on the RDI score undergo repeat breast imaging after six cycles of T-DXd. Patients with iCR after six cycles of T-DXd, regardless of RDI score, proceed to surgery. Patients who have a low/intermediate chance of pCR based on the RDI score and residual disease on imaging after six cycles of T-DXd will undergo either further systemic therapy or proceed to surgery (at the discretion of their treating physician). Regardless of whether the patient will proceed to surgery or undergo further systemic therapy the maximum number of cycles of T-DXd is six cycles. Based on the surgical specimen, patients who achieve a pCR will undergo further trastuzumab post-operatively as per standard of care to complete a total of 52 weeks of systemic therapy from the first cycle of T-DXd. Patients with residual disease at surgery will receive adjuvant chemotherapy as decided by the treating physician. The study design is illustrated in Fig. 1.

### Patients

The main inclusion criteria for this study are as follows: newly diagnosed histologically confirmed HER2-positive breast cancer, age ≥ 18 years, stage 2–3 breast cancer, no prior therapy received for breast cancer, ECOG performance status 0–1 and adequate organ function. Patients with lung-specific intercurrent clinically significant illnesses, any autoimmune or inflammatory disorders with pulmonary involvement or prior pneumonectomy are excluded. Key inclusion and exclusion criteria are detailed in Table 1.

**Table 1:** Key eligibility criteria

Key inclusion criteria	Key exclusion criteria
1. Histologically confirmed HER2-positive breast cancer; 2. Women and men ≥ 18 years of age; 3. Stage 2–3 breast cancer; 4. No prior therapy for breast cancer; 5. ECOG performance status 0–1; 6. Availability of archival tumour biopsy tissue at screening; 7. Left ventricular ejection fraction (LVEF) ≥ 50%, as determined by ECHO or MUGA 8. Adequate haematologic, hepatic and renal laboratory values (collected ≤ 14 days before registration): a. Absolute neutrophil count (ANC) ≥ 1.5 × 10^9^/L b. Platelet count ≥ 100 × 10^9^/L c. Haemoglobin ≥ 9.0 g/dL d. Alanine aminotransferase (ALT) and aspartate aminotransferase (AST) ≤ 3 × upper limit of normal (ULN) e. Total bilirubin ≤ 1.5xULN or < 3 × ULN in the presence of documented Gilbert’s syndrome f. Serum albumin ≥ 25 g/L g. Creatinine clearance (CrCL) ≥ 30 ml/min h. Prothrombin time and either partial thromboplastin or activated partial thromboplastin time ≤ 1.5 × ULN i. No bloods transfusions or granulocyte-colony stimulating factor within 1 week prior to treatment; 9. Evidence of post-menopausal status or negative serum pregnancy test for females of childbearing potential	1. Known metastatic or stage 4 breast cancer; 2. Unstable angina, new-onset angina (≤ 3 months), myocardial infarction < 6 months before registration, symptomatic congestive heart failure (NYHA class II to IV); 3. Corrected QT interval prolongation to > 470 ms (females) or > 450 ms (males) based on screening 12-lead ECG; 4. Uncontrolled arterial hypertension despite optimal medical management (per investigator’s option); 5. Arterial or venous thrombotic or embolic events such as cerebrovascular accident (including transient ischaemic attacks), deep venous thrombosis or pulmonary embolism within 3 months before registration; 6. Non-healing wound, ulcer or bone fracture; 7. Active, clinically serious infections > CTCAE grade 2 (CTCAE v5.0) requiring IV antibiotics, antivirals or antifungals; 8. Patients with evidence or history of bleeding diathesis. Any haemorrhage or bleeding event ≥ CTCAE grade 3 within 4 weeks prior to the start of treatment; 9. Active primary immunodeficiency, known HIV, or active hepatitis B or C infection; 10. History of (non-infectious) interstitial lung disease (ILD)/pneumonitis that required steroids, current ILD/pneumonitis, suspected ILD/pneumonitis that cannot be ruled out by imaging at screening; 11. Lung criteria: a. Lung-specific intercurrent clinically significant illness, including any underlying pulmonary disorder b. Any autoimmune, connective tissue or inflammatory disorders where there is documented, or a suspicion of pulmonary involvement at time of screening c. Prior pneumonectomy (complete); 12. Pregnant or breastfeeding female, unwillingness to use contraceptive measures in males and females; 13. Concomitant use of prohibited medications; 14. Known hypersensitivity to the test drug, test drug class, or excipients in the formulation; 15. Any illness or medical condition that is unstable or could jeopardise the safety of patients and their compliance in the study

*Abbreviations*: *HER2* Human epidermal growth factor 2, *ECOG* Eastern Cooperative Oncology Group, *ECHO* Echocardiogram, *MUGA* Multigated acquisition scan, *ULN* Upper limit of normal, *NYHA* New York Heart Association, *CTCAE* Common Terminology Criteria for Adverse Events, *HIV* Human immunodeficiency virus

### Treatment

Trastuzumab deruxtecan: 5.4 mg/kg administered as an intravenous infusion on day 1, every 3 weeks.

Eligible patients will receive a total of either 4 or 6 cycles of T-DXd. Dose modifications and treatment interruptions are allowed and must be done according to the guidelines in the summary of product characteristics [15]. If treatment has been delayed for more than 4 weeks from the planned date of administration, the patient is withdrawn from the study treatment.

### Objectives

#### Primary objective

To evaluate the efficacy of T-DXd in the neo-adjuvant treatment of HER2 positive breast cancer using pathological complete response (pCR) as the primary endpoint.

#### Secondary objectives

1. To determine the Event-free survival (EFS) and Overall survival (OS) of patients treated with only T-DXd and trastuzumab.
2. To determine EFS and OS of patients treated with systemic therapy other than trastuzumab in addition to T-DXd.
3. To determine EFS and OS of the entire study population.
4. To compare EFS and OS between patients achieving vs not achieving pCR at surgery.
5. To study molecular evolution of tumours during treatment and to develop a biomarker panel that optimises prediction of the pCR.
6. To determine the percentage of patients who achieve pCR after only 4 cycles of T-DXd.
7. To explore the performance metrics (Negative Predictive Value (NPV), Positive Predictive Value (PPV), sensitivity, specificity) for prediction pCR of RDI, imaging and tomosynthesis biopsy, alone and in combination.

### Data collection and management

Study data will be handled confidentially. The study data will be collected using electronic Case Report Forms (eCRFs). Clinical trial data will be entered by authorised site personnel into the electronic data capture system. The investigator must maintain accurate documentation (source data) that supports the information entered in the eCRF. The investigator is responsible for verifying that data entries are accurate and correct by signing the eCRF. All data will be reviewed for completeness and logical consistency queries will be generated via the electronic data capture system to correct or clarify data or request missing information. The designated site staff will be required to respond to these queries in accordance with data entry and data query timelines for the study. Ongoing source data verification will be performed to confirm that data entered into the eCRF by authorised site personnel are accurate, complete, and verifiable from source documents; that the safety and rights of participants are being protected; and that the study is being conducted in accordance with the currently approved protocol and any other study agreements, ICH GCP, and all applicable regulatory requirements. A Project Management File (PMF) will be maintained for the duration of the study.

### Safety and monitoring

All adverse events will be reported routinely on the eCRF. Intensity for each adverse event will be determined by using the current version 5.0 of the National Cancer Institute (NCI) Common Terminology Criteria for Adverse Events (CTCAE) [16], wherever possible. In cases where these criteria do not apply, intensity should be defined according to the general clinical description of grades as per CTCAE. Adverse events of special interest (AESIs) and serious adverse events (SAEs) will be reported to the sponsor within 24 h of the study site becoming aware of the event. Suspected Unexpected Serious Adverse Reactions (SUSARs) will be reported to the Ethics Committee, EudraVigilance and local regulatory authorities where required. The study will be reviewed on a regular basis by the Safety Monitoring Committee. The Safety Monitoring Committee will report its assessment of the continuing benefit-risk of the study and any recommended actions to the Chief Investigator and the Clinical Lead for consideration and appropriate action.

### Biomarker analysis

Tumour tissue will be collected from the baseline biopsy and surgical pathological specimen. This tissue will be used for DNA/RNA sequencing and proteomic analysis. A fresh tumour tissue biopsy will also be collected after completing 2 cycles of therapy, which will be used to calculate the patient’s RDI score. Patient blood samples will be collected at screening and prior to each cycle of treatment. These samples will be used for circulating tumour DNA and RNA analysis and protein biomarker analysis. This translational analysis aims to identify clinically relevant biomarkers to predict benefit or resistance to treatment, and to facilitate future studies.

### Statistical methods

Sample size calculation was performed for the primary outcome. The primary outcome is the percentage of patients who are successfully treated (achieve pCR at surgery) with only T-DXd and trastuzumab. The preliminary data for the statistical design of our study came from our prior JNCI publication [13]. In this publication, the proportion of HER2 + tumours that were RDI score high with neoadjuvant TCH chemo (6 cycles total) was 41%. The pCR rate with a high RDI score was 83% and the pCR rate with a non-high RDI score was 10% [13]. The former percentage was rounded down a little (on the conservative side), and the latter was rounded up to 20% to allow for the effect of the receipt of optional additional preoperative chemotherapy for the purposes of the design of our study herein. There are no pCR data with T-DXd yet. The pCR rate with T-DM1 (four cycles) in the German ADAPT trial [17] was 41%. These are the data points that we used as a foundation for the statistical design of our trial herein.

Given expected pCR rates of 80% and 20% for the RDI high and non-RDI high groups respectively, the expected pCR rate after surgery for the study as a whole is 44%. Because reported pCR rates in unselected patients with standard HER2 directed neoadjuvant therapy are generally up to 50%, 60% is the minimum acceptable limit for likelihood of pCR in patients selected by high RDI score after 2 cycles of neoadjuvant T-DXd. Similarly, the minimum acceptable limit for likelihood of pCR in patients with non-high RDI score is 10%. Given minimum acceptable limits for expected pCR of 60% and 10% for the RDI high and non-RDI high groups respectively, the minimum acceptable limit for expected pCR rate after surgery for the study as a whole is 30%.

For an expected pCR rate after surgery 44%, a sample size of 74 evaluable patients would provide a 95% confidence interval (Wilson method) with a lower bound of 30% for the pCR rate (equivalent to providing statistical power of 80% to test an expected pCR rate of 44% versus an unacceptable pCR of 30%, using a one-sided significance level of 5%). Allowing for a non-evaluable rate of 5–10%, a total sample size of 80 patients should provide 74 evaluable patients, where an evaluable patient is defined as a patient who is evaluated for pCR after surgery. Patients should also have biopsy at Cycle 2 Day 14 ± 4 days for the RDI score assessment, receive a minimum of 4 T-DXd infusions and undergo surgery in order to be considered as evaluable for the primary endpoint.

The primary outcome will be presented along with the 95% exact binomial CI. If the lower bound of the 95% confidence interval exceeds 30%, we can conclude that an unacceptable pCR rate of 30% can be rejected.

EFS and OS will be estimated using the Kaplan–Meier method, with 95% CI for the set of patients treated with only T-DXd followed by trastuzumab. Comparisons between the two sets of patients in DFS and OS will be made using the Log-rank test, presenting the estimated hazard ratio together with 95% CI.

### Ethical aspects and trial registration

The SHAMROCK trial was approved by the National Office for Research Ethics Committee for Clinical Trials (NREC-CT), the NREC for Clinical Investigations of Medical Devices and Performance Studies of In Vitro Diagnostic Medical Devices (NREC-MD), the Health Products Regulatory Authority (HPRA) and the HPRA Medical Devices (HPRA-MD). The trial was registered in the European drug regulatory affairs Clinical Trials database (Eudra-CT 2022–002485-32, June 30, 2022) and ClinicalTrials.gov (ClinicalTrials.gov Identifier: NCT05710666).

## Discussion

Neoadjuvant therapy is the current standard of care for treating ≥ T2 or node-positive HER2-positive breast cancer [18]. Treatment generally consists of a combination of dual HER2 blockade with trastuzumab and pertuzumab, together with a chemotherapy backbone, based on results of the NeoSphere trial [1]. Established neoadjuvant chemotherapy regimens include docetaxel-carboplatin or an anthracycline-taxane course, with dual antibody blockade. While these regimens have been highly effective, they are associated with significant side effects and toxicity, including febrile neutropenia and cardiotoxicity. The TRAIN-2 trial reported that there is no increased efficacy with an anthracycline containing regimen compared to a taxane-platinum regimen, while cardiac toxicity was higher in the anthracycline containing arm [19]. More recently, the possibility of replacing systemic chemotherapy with a targeted agent associated with less toxicity has been investigated. In the phase II KRISTINE study, neoadjuvant T-DM1 plus pertuzumab was compared to standard chemotherapy with dual HER2-blockade. While the pCR rate was significantly higher with conventional systemic chemotherapy (44.4% vs 55.7%), the rate of grade 3 or greater adverse events was significantly lower in the T-DM1 plus pertuzumab arm (31.8% vs 67.7%) [5].

T-DXd is an ADC which has shown great efficacy in HER2-positive breast cancer [20]. It has a significantly higher drug-to-antibody ratio than T-DM1, however the stability of the linker appears to improve efficacy, without marked side effects [21]. The cytotoxic payload, DXd, is also cell membrane permeable, which enables elimination of both target tumor cells and the surrounding tumor cells, known as the bystander antitumor effect [22]. T-DXd showed a substantial benefit in patients with HER2-positive metastatic breast cancer who had received prior treatment with T-DM1 in the DESTINY-Breast01 trial [8]. In DESTINY-Breast03, a significantly higher progression free survival (PFS) and overall response rate (ORR) was reported with T-DXd compared to T-DM1 in HER2-positivie metastatic breast cancer previously treated with trastuzumab and a taxane [23]. Furthermore, T-DXd was found to be superior to the physicians choice of chemotherapy in patients who had progressed on T-DM1, showing that it could overcome resistance to another ADC [9].

Based on the promising results of T-DXd in the advanced/metastatic setting, adjuvant and neoadjuvant trials were designed. Adjuvant T-DXd is being investigated in the ongoing DESTINY-Breast05 trial, comparing T-DXd to T-DM1 in patients with residual invasive HER2-positive breast cancer following neoadjuvant therapy [24]. Neoadjuvant T-DXd is also currently being evaluated in the DESTINY-Breast11 trial in locally advanced or inflammatory HER2-positive breast cancer [25]. The SHAMROCK study will investigate neoadjuvant T-DXd in patients with early stage HER2-positive breast cancer, incorporating therapy escalation and de-escalation strategies as described above.

The SHAMROCK study will use pCR as the primary endpoint. pCR at surgery is correlated with favourable patient outcomes, particularly in HER2-positive, hormone-receptor negative (HR-) breast cancer, as shown by the CTNeoBC pooled analysis [3]. Numerous additional meta-analyses have subsequently supported the value of pCR as an informative surrogate biomarker for improved survival in HER2-positive breast cancer [26–28]. To optimise pCR rates, the RDI score will be used as a biomarker for on-treatment tissue to guide neoadjuvant chemotherapy escalation or de-escalation. The RDI score will be performed on a biopsy after 2 cycles of T-DXd (cycle 2 day 14 ± 4), which will provide an opportunity to adapt treatment and potentially avoid additional cycles of ineffective therapy.

This phase II study will investigate neoadjuvant T-DXd in early stage HER2-positive breast cancer. We hypothesise that this novel adaptive trial design will achieve high pCR rates and avoid unnecessary toxicity, while also exploring predictive and prognostic biomarkers for treatment.

## Trial status

The SHAMROCK study is a nationwide multicentre phase 2, open-label, single-arm trial. The study opened for accrual on 26th October 2023.

## Data Availability

Not applicable.

## Abbreviations

ADC: Antibody–drug conjugate
AESI: Adverse events of special interest
CI: Confidence interval
CTCAE: Common Terminology Criteria for Adverse Events
DFS: Disease-free survival
eCRF: Electronic case report form
EFS: Event-free survival
HER2: Human epidermal growth factor 2
HPRA: Health Products Regulatory Authority
HR: Hormone-receptor
ICH GCP: International Council for Harmonisation of Technical Requirements for Pharmaceuticals for Human Use Guideline for Good Clinical Practice
iCR: Imaging complete response
NCI: National Cancer Institute
NREC: National Office for Research Ethics Committee
ORR: Overall response rate
OS: Overall survival
pCR: Pathological complete response
PFS: Progression-free survival
PMF: Project Management File
RDI: RNA disruption index
RNA: Ribonucleic acid
SAE: Serious adverse event
SUSAR: Suspected Unexpected Serious Adverse Reaction
T-DM1: Trastuzumab emtansine
T-DXd: Trastuzumab-deruxtecan
